# Pathway Markers for Pro-resolving Lipid Mediators in Maternal and Umbilical Cord Blood: A Secondary Analysis of the Mothers, Omega-3, and Mental Health Study

**DOI:** 10.3389/fphar.2016.00274

**Published:** 2016-09-07

**Authors:** Ellen L. Mozurkewich, Matthew Greenwood, Chelsea Clinton, Deborah Berman, Vivian Romero, Zora Djuric, Clifford Qualls, Karsten Gronert

**Affiliations:** ^1^Obstetrics and Gynecology, University of New MexicoAlbuquerque, NM, USA; ^2^Obstetrics and Gynecology, University of MichiganAnn Arbor, MI, USA; ^3^Vision Science Program, Infectious Diseases and Immunity Program, School of Optometry, University of California at BerkeleyBerkeley, CA, USA; ^4^Obstetrics of Gynecology, Duke UniversityDurham, NC, USA; ^5^Obstetrics and Gynecology, Michigan State UniversityGrand Rapids, MI, USA; ^6^Family Medicine, University of MichiganAnn Arbor, MI, USA; ^7^Clinical and Translational Sciences Center, University of New MexicoAlbuquerque, NM, USA

**Keywords:** resolvins, EPA, DHA

## Abstract

The omega-3 fatty acids docosahexaenoic acid (DHA) and eicosapentaenoic acid (EPA) are precursors to immune regulatory and specialized pro-resolving mediators (SPM) of inflammation termed resolvins, maresins, and protectins. Evidence for lipid mediator formation *in vivo* can be gained through evaluation of their 5-lipoxygenase (LOX) and 15-LOX metabolic pathway precursors and downstream metabolites. We performed a secondary blood sample analysis from 60 participants in the Mothers, Omega-3, and Mental Health study to determine whether SPM and SPM precursors are augmented by dietary EPA- and DHA-rich fish oil supplementation compared to soy oil placebo. We also aimed to study whether SPM and their precursors differ in early and late pregnancy or between maternal and umbilical cord blood. We found that compared to placebo supplementation, EPA- and DHA-rich fish oil supplementation increased SPM precursor 17-hydroxy docosahexaenoic acid (17-HDHA) concentrations in maternal and umbilical cord blood (*P* = 0.02). We found that the D-series resolvin pathway marker 17-HDHA increased significantly between enrollment and late pregnancy (*P* = 0.049). Levels of both 14-HDHA, a maresin pathway marker, and 17-HDHA were significantly greater in umbilical cord blood than in maternal blood (*P* < 0.001, both).

## Introduction

A variety of putative health benefits have been attributed to omega-3 fatty acids, including prevention of cardiovascular disease, reduction of chronic inflammation, reduction in depressive symptoms, and prevention of insulin resistance (Delarue et al., 2006; Gonzalez-Periz et al., 2006; Hassan and Gronert, 2009). In pregnancy, omega-3 fatty acid supplementation has been shown to reduce early preterm births (<32–24 weeks; Makrides et al., 2006, 2010; Szajewska et al., 2006; Horvath et al., 2007) as well as to decrease risk for infant admission to a neonatal intensive care unit (Makrides et al., 2010).

Omega-3 fatty acids were originally thought to exert these beneficial effects by competitively inhibiting formation of pro-inflammatory arachidonic acid-derived eicosanoids (Gonzalez-Periz et al., 2006). However, more recently, omega-3 fatty acids have been shown to be the precursors to novel classes of specialized pro-resolving mediators (SPM) that are formed by lipoxygenase (LOX) or cyclooxygenase enzymes analogous to the formation of the arachidonic acid-derived SPM class of lipoxins. These ω-3 polyunsaturated fatty acid (PUFA)-derived mediators are classified as E-series resolvins [eicosapentaenoic acid (EPA) metabolites], D-series resolvins [docosahexaenoic acid (DHA) metabolites], maresins (DHA metabolites), and protectins (DHA metabolites) (Fredman and Serhan, 2011; Serhan, 2014). *In vitro* and *in vivo* experimental evidence strongly suggests that increased resolvin, maresin, and protectin formation after dietary supplementation may explain many of the observed health benefits of dietary ω-3 PUFA supplementation (Gronert, 2008; Grenon et al., 2015; Murphy, 2015; Serhan et al., 2015).

**FIGURE 1:**
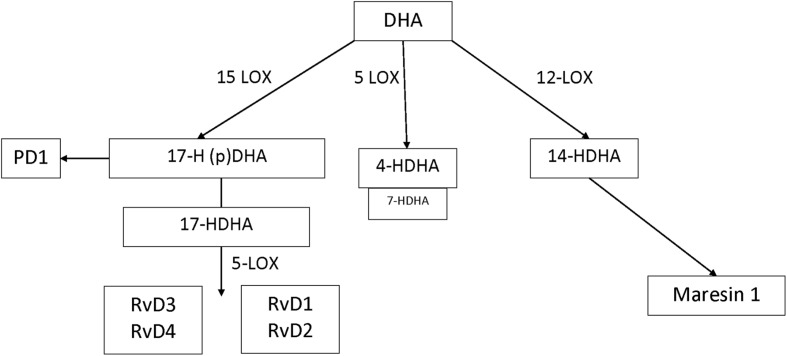
**DHA metabolome**.

In humans, many of the prominent DHA-derived resolvins are primarily synthesized through the interaction of 15-LOX with 5-LOX. A key intermediate is the 15-LOX product 17-hydroperoxy-DHA; its metabolite 17-hydroxy docosahexaenoic acid (17-HDHA) is pathway marker for the D-series resolvins. 17-Hydroperoxy-DHA is also the rate-limiting intermediate for the formation of protectin D1, which is 15-LOX product and that does not require 5-LOX (Serhan and Petasis, 2011). The primary hematologic cell types that generate 17S-HDHA are eosinophils (high expression) (Miyata et al., 2013) monocytes (low expression and high expression in macrophages) and polymorphonuclear leukocytes (low expression but inducible during resolution) (Serhan, 2014). By contrast, DHA-derived maresins are formed via the 12-LOX intermediate 14-hydroperoxy-DHA and further conversion by 5-LOX. 14-HDHA is the pathway marker for maresin formation and 12-LOX activity, an enzyme that is highly expressed in platelets and at lower levels in macrophages (Barden et al., 2014). 4-HDHA is another 5-LOX metabolite, that in animal models has been shown to mediate the antiangiogenic effect of omega-3 fatty acids (Sapieha et al., 2011) (**Figure 1**). By contrast, the EPA-derived resolvins are primarily synthesized through the cytochrome P450 intermediate 18S-hydroxyeicosapentaenoic acid (18S-HEPE), that is further converted by 5-LOX (Barden et al., 2014).

In healthy, non-pregnant volunteers, endogenous formation of maresins, resolvins, and protectins may be amplified by dietary omega-3 fatty acid supplementation (Fredman and Serhan, 2011; Gomolka et al., 2011). Due to the short half-life and rapid elimination of the potent resolvins, protectins, and maresins, direct quantification and detection of SPM in archived tissue samples may be challenging (Psychogios et al., 2011; Skarke et al., 2015). However, their specific metabolic intermediates and pathway markers have established activation of SPM pathways and their formation *in vivo* both in humans and animal models of inflammatory diseases. In particular 18-HEPE, 17-HDHA and 14-HDHA are established markers for the activation of the E-series resolvin, D-series resolvin and maresin biosynthetic pathways, respectively (Weylandt et al., 2012; Barden et al., 2014).

Endogenous synthesis of specialized pro-resolving lipid mediators and their pathway markers in maternal and fetal serum has not been previously studied. The proposed study represents a secondary analysis of stored maternal and fetal (cord blood) serum samples from subjects who participated in a double blind randomized controlled trial comparing EPA-rich fish oil (1060 mg EPA plus 274 mg DHA) and DHA-rich fish oil (900 mg DHA plus 180 mg EPA) supplementation with soy oil placebo for prevention of perinatal depression among women at risk (Mozurkewich et al., 2013). We performed this ancillary study in order to compare maternal and neonatal activity of resolvin, protectin, and maresin pathway markers (RPM). We also aimed to study whether RPM differed in maternal and umbilical cord blood from subjects who were supplemented with EPA-rich fish oil, DHA-rich fish oil, versus placebo. We hypothesized that pathway markers of DHA-derived resolvins and protectins would be enhanced in umbilical cord blood compared to maternal blood, due to the preferential transplacental transport of DHA in late pregnancy. We further hypothesized that dietary supplementation with EPA- and DHA-rich fish oils would amplify resolvin, protectin, and/or maresin pathway activity compared with placebo supplementation.

### Ethics

The parent trial was registered at Clinicaltrials.gov at NCT00711971. This parent study and this secondary analysis were approved by the University of Michigan Institutional Review Board at HUM00004684. This secondary analysis was deemed exempt by the University of New Mexico Health Sciences Center Human Research Protection Organization where the data analyses were carried out.

## Materials and Methods

This study was a secondary analysis using LC/MS/MS (liquid chromatography/mass spectroscopy/mass spectroscopy) of stored plasma samples that were collected as part of a prospective, blinded randomized controlled trial of fish oil supplementation for prevention of depressive symptoms among women at risk (Mozurkewich et al., 2013). The parent trial was carried out at two medical centers in southeastern Michigan, the University of Michigan Health System and St. Joseph Mercy Health System. One hundred twenty-six subjects, of whom 118 completed the trial, enrolled in the study between October 2008 and May 2011. Potential subjects were excluded if they were eating more than two fish meals weekly, or if they were taking omega-3 fatty acid supplements, including prenatal vitamins with DHA (Mozurkewich et al., 2013). They were randomly assigned to receive EPA rich fish oil (1060 mg EPA plus 274 mg DHA), DHA rich fish oil (900 mg DHA plus 180 mg EPA), or soy oil placebo, and were followed longitudinally through pregnancy from their enrollment at 12–20 weeks’ gestation, through 6 weeks postpartum. Maternal blood was drawn at enrollment (visit 1) and again at 34–36 weeks gestation (visit 3). Umbilical cord blood was obtained after the delivery of the infants born to mothers enrolled in the study (visit 4). Serum aliquots prepared from each blood collection and were stored at -70°C (Mozurkewich et al., 2013). All samples were processed and stored within 12 h. Results of the blood sample analyses for fatty acids have been previously described (Mozurkewich et al., 2013).

This exploratory study involved a subgroup analysis of serum samples collected from 60 mother–infant pairs of study participants. Due to cost considerations, it was not possible to perform analyses for the whole study cohort. Samples for analysis were selected based on availability of complete sample sets and adherence to the protocol as ascertained by capsule counts.

Stored samples were analyzed by LC/MS/MS based lipidomic analysis to quantify levels of ω-3 PUFA-derived SPM. In brief, 400 pg of class specific deuterated internal standards prostaglandin E2 (PGE2-d4), lipoxin A4 (LXA4-d5), leukotriene B4 (LTB4-d4), 15(S)-hydroxyeicosatetraenoic acid [15(S)-HETE-d8], eicosatetraenoic acid (arachidonic acid-d8) and docosahexaenoic acid (DHA-d5) were added to each sample prior to processing and extraction to calculate the recovery of specific classes of oxygenated fatty acids. Lipid antacids were extracted by solid phase with SampliQ ODS-C18 cartridges (Agilent Technologies). Eicosanoids and docosanoids were identified and quantified by LC/MS/MS-based lipidomics based on our published methods (Hassan and Gronert, 2009). Extracted samples were analyzed by a triple-quadrupole linear ion trap LC/MS/MS system (MDS SCIEX 3200 QTRAP) equipped with a Kinetex C18 mini-bore column. The mobile phase was a gradient of A [water/acetonitrile/acetic acid (72:28:0.01, v:v:v)] and B [isopropanol/acetonitrile (60:40, v:v)] with a 450 μl/min flow rate. MS/MS analyses was performed in negative ion mode and prominent fatty acid metabolites quantified by multiple reaction monitoring (MRM mode) using established and specific transitions as previously described (González-Périz et al., 2009; Hassan and Gronert, 2009; Sapieha et al., 2011; von Moltke et al., 2012; Kalish et al., 2013; Pruss et al., 2013). Calibration curves (1–1000 pg) and specific LC retention times for each compound were established with synthetic standards (Cayman Chemical, Ann Arbor, MI, USA). Structures were confirmed for selected autacoids by MS/MS analyses using enhanced product ion mode with appropriate selection of the parent ion in quadrupole 1. EPA, DHA, and arachidonic acid metabolomes were constructed. For inclusion, levels of ω-3 PUFA-derived SPM needed a signal-to-noise ratio of at least 3:1. When the signal-to-noise ratio did not meet this inclusion criteria, we used the lower limits of detection, which were 1 pg/ml for 4-HDHA, 5 pg/ml for 14-HDHA, and 2 pg/ml for 17-HDHA.

### Statistics

For comparison of the baseline characteristics between the three treatment groups, we used analysis of variance (ANOVA). Comparisons for categorical variables were done using Fisher’s exact tests. For other outcome measures computed descriptive statistics reported as mean and standard error of the mean for continuous data and frequency (%) for binary and categorical data. For values below the limits of detection, we imputed the lower limit of detection for the assay used. Data with right-skewed distributions were logarithmically transformed. We analyzed log-transformed resolvin pathway marker concentrations across visits 1, 3, and 4 (with visit 4 representing neonatal cord blood), with repeated measures (RM) ANOVA with “visit” as the repeated factor and the three “groups” (EPA, DHA, and placebo) as the grouping factor. Where there was no difference between individual treatment groups versus placebo, the treatment groups (EPA and DHA) were combined for subsequent RM ANOVA exploratory analysis. In instances in which significance was found by RM ANOVA, further exploratory analyses were carried out in the following manner. *Post hoc* tests of change at visit 3 (post-supplementation) were done by paired *t*-test. *Post hoc* comparisons between groups were done using unpaired *t*-tests. Pearson’s correlation coefficient was computed to describe the relationship between log-transformed serum DHA levels at each study visit and log-transformed 4-HDHA, 14-HDHA, and 17-HDHA. For the purposes of the correlation analyses, the values below the lower limit of detection were removed. An additional correlation analysis was carried out to evaluate the relationship between maternal DHA at 34–36 weeks and cord blood 17-HDHA. A *P*-value of 0.05 was considered statistically significant. We conducted an exploratory analysis of the effect of labor on 17-HDHA formation by comparing log transformed 17-HDHA concentrations in cord blood collected after cesarean section without labor versus cord blood from birth after labor using unpaired *t*-tests.

## Results

The baseline demographic characteristics of the study subjects in the subset chosen for this analysis did not differ significantly between the treatment groups. Capsule compliance was similar between the three groups with subjects in the EPA group having an overall compliance of 69%, subjects in the DHA group with 71% compliance, and subjects in the placebo group with 76% compliance. This difference was not statistically significant (*P* = 0.60, generalized linear models). There was no difference in mean age at screening, gravidity, parity, or history of prior preterm delivery among the treatment groups (ANOVA, all *P* ≥ 0.50) There was no difference in ethnicity or race (Fisher’s exact test, both *P* > 0.35; **Table 1**).

**Table 1:** Demographic table.

	EPA	DHA	Placebo	Significance
*N*	19	21	20	
Age at screening	29.0 ± 5.5	30.4 ± 4.8	30.9 ± 6.1	NS
Gravidity	2.37 ± 1.38	2.33 ± 1.15	2.20 ± 1.91	NS
Parity, term	0.68 ± 0.95	0.90 ± 1.04	0.60 ± 0.88	NS
Preterm	0.105 ± 0.315	0.095 ± 0.301	0.200 ± 0.523	NS
Av. compliance	0.69 ± 0.25	0.71 ± 0.20	0.76 ± 0.22	NS
White	16	17	16	NS

*Av = average; NS = not significant*.

D-series resolvins, E-series resolvin, protectins, and maresins were not consistently detected; peaks were below acceptable signal-to-noise ratio for the 3200 QTRAP MRM-based identification and quantification. The E-series resolvin pathway marker, 18-HEPE, was below detectable limits in 96% of the samples. Thus no further analysis of the EPA metabolome was carried out.

**FIGURE 2:**
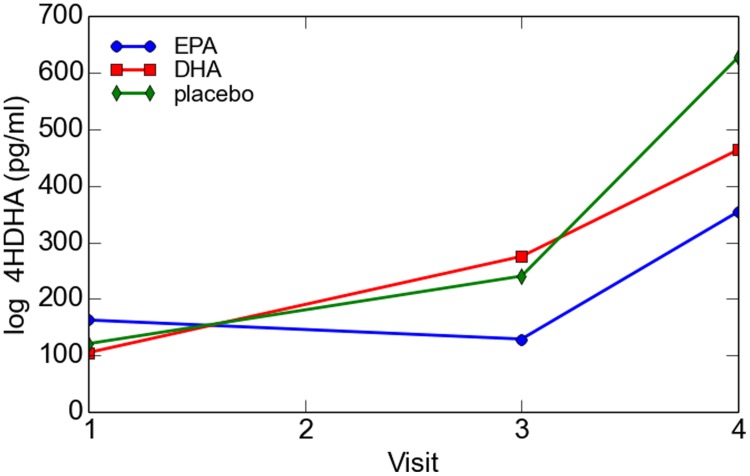
**Maternal and cord blood log 4-HDHA concentration**.

**FIGURE 3:**
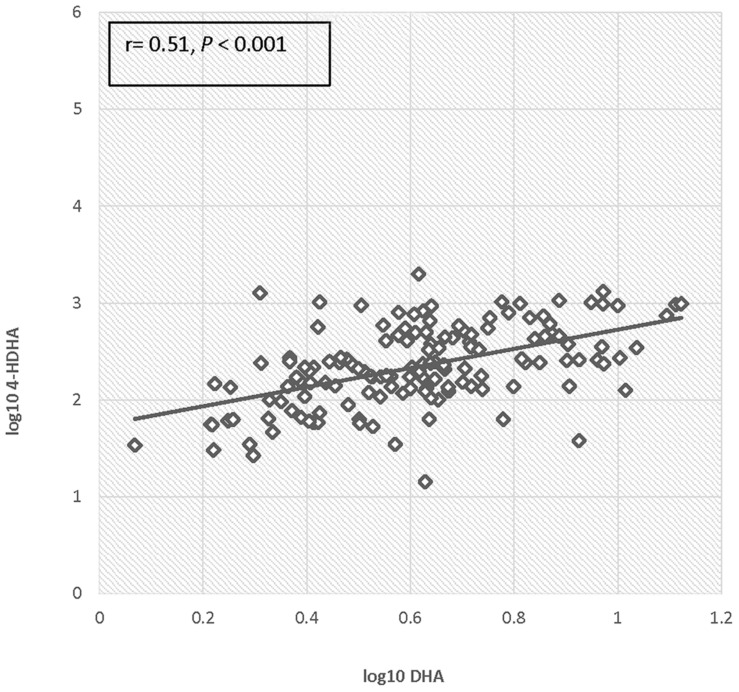
**Correlation of log DHA fraction with log 4-HDHA concentration**.

There was no significant difference in 4-HDHA levels between the groups at study entry or at any subsequent study visit. Log transformed 4-HDHA levels did not significantly differ across the study visits and were not significantly different in the maternal blood versus umbilical cord blood (**Figure 2**). Log transformed 4-HDHA concentrations were significantly correlated with DHA levels (Pearson’s correlation coefficient 4 = 0.51, *P* < 0.001; **Figure 3**).

**FIGURE 4:**
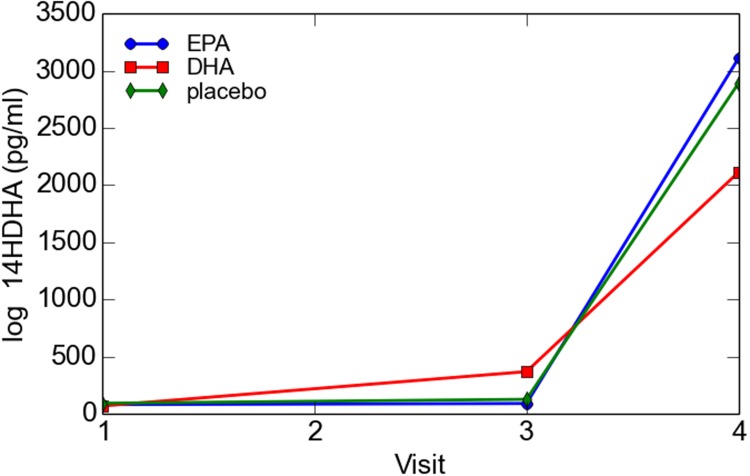
**Maternal and cord blood log 14-HDHA concentrations**.

**FIGURE 5:**
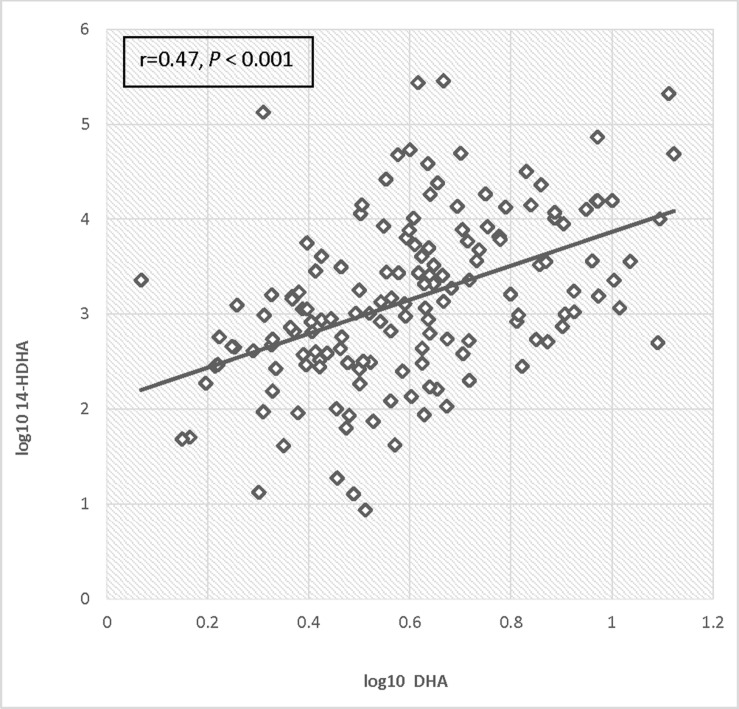
**Correlation of log DHA fraction with log 14-HDHA concentrations**.

Log transformed 14-HDHA did not differ significantly between early and late pregnancy. 14-HDHA levels were significantly increased in umbilical cord blood compared to maternal blood at both maternal blood draw time points (*P* < 0.001). There was no significant treatment effect of EPA- and DHA-rich fish oil supplementation compared to placebo on 14-HDHA levels, in either maternal blood or umbilical cord blood (**Figure 4**). However, in an analysis of combined blood samples from all study visits, 14-HDHA levels were significantly correlated with DHA levels (Pearson’s correlation coefficient 0.47, *P* < 0.001; **Figure 5**).

**FIGURE 6:**
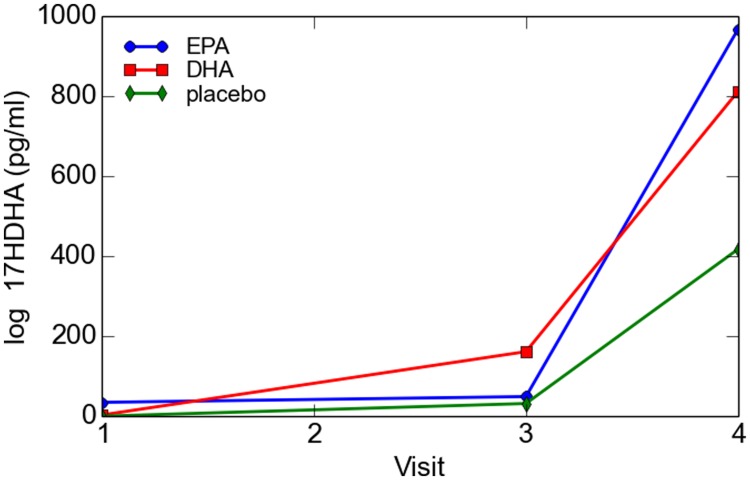
**Maternal and cord blood log 17-HDHA concentrations**.

**FIGURE 7:**
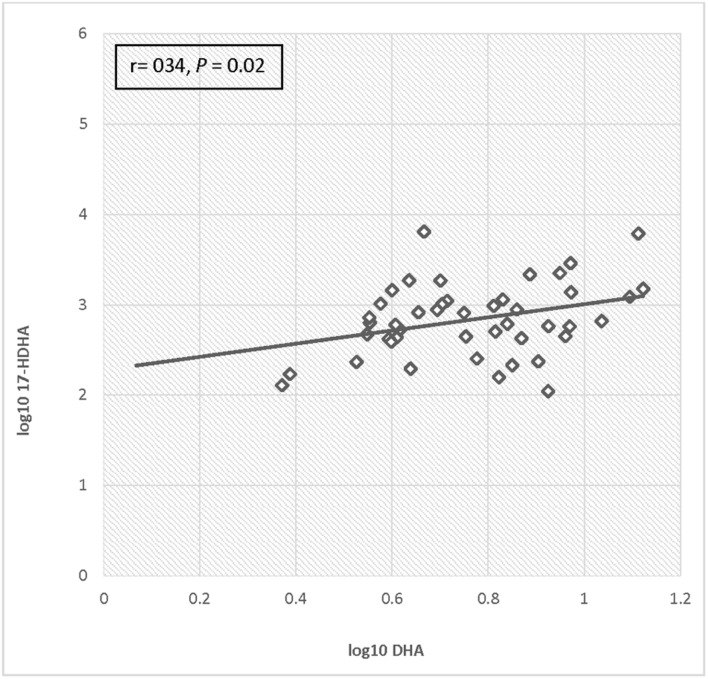
**Correlation of log DHA fraction with log 17-HDHA concentrations**.

For the total cohort, 17-HDHA levels significantly increased in maternal blood between enrollment and 34–36 weeks (*P* = 0.049). Concentrations of 17-HDHA in umbilical cord blood were significantly greater than in maternal blood both at study entry and at 34–36 weeks gestation (*P* < 0.001; **Figure 6**). In the total sample set, 17-HDHA levels were significantly correlated with DHA levels (Pearson’s correlation coefficient 0.34, *P* < 0.02; **Figure 7**).

When the EPA- and DHA-rich fish oil groups were individually compared with placebo using repeated measures modeling, there was a trend toward higher 17-HDHA activity that did not reach significance (*P* = 0.07). By contrast, when the two supplementation groups were combined for the purpose of analysis, 17-HDHA activity was significantly higher in the combined EPA- and DHA-rich fish oil group than in the placebo supplemented group (*P* = 0.02).

However, when the EPA- and DHA-rich oil groups were together compared with placebo at the post-supplementation time points using the *t*-test, there were no significant differences between the treatment groups and placebo at either of the post-supplementation time points. Although there was a trend toward treatment effect for supplementation at visit 3 (*P* = 0.11, *t*-test) this effect was not statistically significant. Likewise, although concentrations of 17-HDHA in umbilical cord blood were higher in the treatment groups as compared to placebo, this trend was not significant (*P* = 0.33). Of interest, cord blood levels of 17-HDHA were 5-fold higher after exposure to labor than in the instance of elective cesarean section, but this difference did not reach statistical significance.

## Discussion

The main finding of our study is that the maresin pathway precursor 14-HDHA and the resolvin pathway marker 17-HDHA were significantly enhanced in cord serum compared to maternal serum in early and late pregnancy. This finding is consistent with the observation of augmentation of DHA percentage in umbilical cord serum compared to maternal serum that we observed in the parent study and that has also been reported in other studies (Mozurkewich et al., 2013; Baack et al., 2015). Our study also suggested a treatment effect for EPA- and DHA-rich fish oil supplementation for the resolvin pathway precursor 17-HDHA, but prospective randomized studies are needed to determine whether resolvins, protectins, and maresins, themselves, are augmented by supplementation (Colas et al., 2014).

Previous studies examining the presence of SPM in adults have looked at subsets with specific diseases, including asthma, Alzheimer’s disease, multiple sclerosis, chronic kidney disease, peripheral artery disease, and rheumatoid arthritis (Serhan, 2014; Grenon et al., 2015; Mas et al., 2016) as well as in unselected adults (Psychogios et al., 2011). In the reproductive sphere, maternal omega-3 fatty acid supplementation with 3.7 g DHA plus EPA was shown to increase resolvin and protectin precursors in human placental tissues; likewise, a high n-3 PUFA diet has been shown to increase SPM precursors in rat placentas (Jones et al., 2013; Keelan et al., 2015). This study differs from these prior evaluations in that it assessed resolvin, protectin, and maresin pathway activity in human umbilical cord blood in relation to maternal dietary supplementation with DHA and EPA.

Our study was limited by the small sample size as well as cost constraints that precluded analysis of the entire trial cohort. Similarly, the sample subset analyzed was not randomly chosen, but was selected based on maternal compliance with supplementation and by availability of complete sample sets. In terms of quantifying ω-3 PUFA-derived SPM our analysis was limited to quantifying DHA-derived resolvin, maresin, and protectin pathway markers, which likely was due to using an LC/MS/MS system with 1–10 pg limits of quantification. The DHA-derived resolvin pathway precursors we evaluated were below the limits of detection in some of our samples, and the precursors of the E-series resolvins were detected in only a few samples. In addition, although several investigations in animal models suggest that SPM may act to inhibit preterm delivery and insulin resistance (González-Périz et al., 2009; Hellmann et al., 2011; Yamashita et al., 2013), our study was not designed to determine whether increased SPM levels after supplementation would confer any clinical benefit.

Another potential weakness of this study is the possibility that autoxidation may have led to higher levels of some pathway markers rather than enzymatic conversion of DHA. Our protocol for the timing of specimen processing and storage was also chosen to minimize autoxidation artifacts. The patients and treatment-specific lipidomic profiles and differing levels of the monohydroxy metabolites 4-HDHA, 14-HDHA, and 17-HDHA in each sample also suggest that autoxidation did not significantly contribute to the overall lipidomic profiles.

It is important to note that resolvins, protectins, and maresins are produced in self-limited inflammatory leukocyte-rich exudates and are temporally regulated during the resolution phase of acute inflammation (Serhan, 2014). This may explain the increase in cord blood levels of 17-HDHA after exposure to labor, which requires activation of inflammatory pathways, compared to elective cesarean section (Gonzalez et al., 2011). Lipid mediators have also been found to be 10–100 times higher in serum compared to plasma (Colas et al., 2014). Finally, higher levels of SPMs were recently reported in human milk when compared to those observed in peripheral blood samples of healthy individuals (Weiss et al., 2013).

Thus, future research examining SPM pathway activity in serum during phases of acute inflammation, such as normal and abnormal labor would be of great interest. We have previously reported that DHA-rich fish oil supplementation increased DHA levels in maternal and umbilical cord blood, compared to placebo (Mozurkewich et al., 2013). While we did not demonstrate any effect of fish oil dietary supplementation on SPM themselves, the trend toward treatment effect of supplementation on resolvin pathway markers in our small sample should lead to larger studies evaluating the effect of supplementation on SPM and any associations with clinical outcomes. Further, given the reported high concentration of SPMs in human milk, it would be beneficial to collect the serum of breastfed neonates of mothers supplemented with omega-3 fatty acids for examination of newborn host defense and development. Continued research on specialized SPM profiling is also needed because human resolution phenotypes have only recently been developed (Morris et al., 2010). Now that studies have proven the presence of SPMs in adult and fetal tissue, as well as their potential for modulation, the stage has been set for further identification of SPM function and relationship to nutrition and disease. Specifically, further research is needed to elucidate the potential benefits for prenatal augmentation of RPM in the fetal compartment.

## Author Contributions

EM designed and carried out the parent study and this secondary analysis and wrote the paper. CC, DB, and VR helped to carry out the study and wrote the paper. MG carried out the blood sample analyses described in this analysis. KG designed this secondary analysis, carried out blood sample analyses, and wrote the paper. ZD designed and carried out the parent study and wrote the paper. CQ carried out the statistical analyses and wrote the paper.

## Conflict of Interest Statement

The authors declare that the research was conducted in the absence of any commercial or financial relationships that could be construed as a potential conflict of interest. The reviewer AF and handling Editor declared their shared affiliation, and the handling Editor states that the process nevertheless met the standards of a fair and objective review.
